# The Wind Energy Potential of Kurdistan, Iran

**DOI:** 10.1155/2014/323620

**Published:** 2014-10-27

**Authors:** Farzad Arefi, Jamal Moshtagh, Mohammad Moradi

**Affiliations:** ^1^Department of Power Electrical Engineering, College of Engineering, Islamic Azad University, Kurdistan Science and Research Branch, Sanandaj 6614996164, Iran; ^2^Electrical Engineering Department, Kurdistan University, Pasdaran Avenue, Sanandaj, Iran; ^3^Engineering Faculty, Razi University, Abrisham Avenue, Kermanshah, Iran

## Abstract

In the current work by using statistical methods and available software, the wind energy assessment of prone regions for installation of wind turbines in, Qorveh, has been investigated. Information was obtained from weather stations of Baneh, Bijar, Zarina, Saqez, Sanandaj, Qorveh, and Marivan. The monthly average and maximum of wind speed were investigated between the years 2000–2010 and the related curves were drawn. The Golobad curve (direction and percentage of dominant wind and calm wind as monthly rate) between the years 1997–2000 was analyzed and drawn with plot software. The ten-minute speed (at 10, 30, and 60 m height) and direction (at 37.5 and 10 m height) wind data were collected from weather stations of Iranian new energy organization. The wind speed distribution during one year was evaluated by using Weibull probability density function (two-parametrical), and the Weibull curve histograms were drawn by MATLAB software. According to the average wind speed of stations and technical specifications of the types of turbines, the suitable wind turbine for the station was selected. Finally, the Divandareh and Qorveh sites with favorable potential were considered for installation of wind turbines and construction of wind farms.

## 1. Introduction

Because of the population growth and consequently a dramatic increase in the demand for energies, the constraints of fossil and nonrenewable resources, energy crisis, prices fluctuation, and destructive effects originating from burning of energy resources and due to an increase in environmental pollution that severely threatens human life, on the one hand, the clean, nonpolluting, and great potential of renewable energy opened up an expansive vista in the utilization of the solar, wind, water, and wave energy. The global potential for production of energy from wind has been investigated. In general investigation, the theoretical and exploitable potential estimated of wind energy in the world were about 10^5^ EJ (each EJ is equivalent to 10^18^ J or is equivalent to 1.634 × 10^5^ barrels of petroleum) and 110 EJ (equivalent to 1.7974 × 10^10^ barrels of petroleum), respectively [1].

Like other developing countries, Iran has been encountered with significant challenges in energy and environmental policies. Economic growth in Iran depends on electricity consumption.

The energy production and also consumption have increased in Iran in recent years due to its rapid economic growth [2].

The necessity of renewable energy in Iran can be categorized in three main issues: (a) environmental pollution, (b) limited fossil resources, and (c) more oil and gas export. The most important environmental problem that Iran faces is air pollution and one of the main sources ofair pollutionis burningfossil fuels [3]. Another problem related to fossil fuels is that the fossil fuels are nonrenewable. They are limitedin supply and will one day be depleted. There is no escaping from this conclusion. Another issue that motivates the use of renewable energy in Iran is the price of fuel for producing electric energy, and exportation, since 80% of Iran's revenue is based on oil and gas exportation [3, 4]. Therefore the government is paying to subsidize electric energy production. The harmful effects of fossil fuels, such as air pollution, changes in rainfall, and climate, the high price of fossil fuels, and limited fossil resources force the researcher to think about the need for renewable energy seriously [3]. Hence, wind energy appears as a clean and good solution to cope with a great part of this energy demand [5].

The horizontal movement of air parallel to the earth's surface is a measure of the wind in both direction and magnitude. Wind energy is directly related to wind speed and other meteorological factors. For production of electricity by wind turbines, convenient area selection for greater efficiency is important and necessary [6]. For evaluation of wind energy potential, the sources of wind speed and energy change with time and other factors must be taken into account carefully and correctly [7].

Iran with the unique climate and geographical position in the middle Asia between warm and temperate weather areas of Asia, Europe, Africa, the Indian Ocean, and the Atlantic Ocean has a considerable wind blowing potential and is potentially one of the best regions for utilization of most alternative sources of renewable energies. Lack of compressive study of wind energy potential in Kurdistan for its exploitation is clear [3, 7, 8].

To provide clear image of the wind energy potential in our selected area (Kurdistan-Qorveh), the scientific investigations and using the experiences of precursor countries in wind industry are needed. In this research, the wind resource potentials with electricity production ability in Qorveh (for installation of wind turbines) by applied methods and geographical information are discussed.

## 2. Experimental and Theoretical

In this study, the wind energy potential of Qorveh was studied. The weather station data for speed and direction of wind were obtained from Iran Meteorological Organization (since its establishment up to 2005) and Kurdistan Meteorological site (after 2005).

The monthly and annual average and maximum speed for Qorveh station in period of 2000–2010 were analyzed. The statistical processing of wind speed and wind direction in synoptic station of Kurdistan was carried out. The ranking of meteorological stations of the country in period of 10 years (1993–2002) was gathered for calibration of wind map to have a reliable picture of wind regime. Wind Rose model and diagram providing the dominant wind in an area, which can be determined, were used to the recognition of wind speed and direction status in different times in a certain place. The WRPLOT VIEW software has been used to draw a Wind Rose diagram [9]. Suitable turbine from Gamesa (G58-850) and Suzlon (S64-650) companies was selected. The Logarithmic Law (log-law) was used for theoretical calculation of wind speed at different heights.

Feasibility study includes wind blowing status, site evaluation, and selection, model selection of wind turbine. Some of the important factors of wind blowing for a site include: wind speed, wind speed distribution, wind direction distribution, daily pattern of wind speed, and yearly pattern of wind speed [9].

### 2.1. The Logarithmic Law (Log-Law) for Theoretical Calculation of Wind Speed

The log-law (see (1)) is a relationship between the wind speeds at one height and those at another (reference height) [10]. Regional status of the studied area is important in aspect of surface roughness and atmospheric stability. Consider

(1) $$\begin{array}{c} \frac{\;\ln\left(h/Z_{0}\right)}{\ln\left(h_{\text{ref}}/Z_{0}\right)\;\;}=\frac{V}{V_{\text{ref}}}, \end{array}$$

where *V* (m/s) is the wind speed at height *h* (m), *V*_ref_ (m/s) is the known wind speed at a reference height *h*_ref_ (m) and *Z*_0_ (m) is the surface roughness length, which is determined according to Table 1, considering the types of land surfaces [10].

### 2.2. Power Law for Wind Speed Calculation

The power law is defined as [11, 12]

(2) $$\begin{array}{c} {\left(\frac{h}{h_{\text{ref}}}\right)}^{\alpha }=\frac{V}{V_{\text{ref}}}, \end{array}$$

where *V* (m/s) is the speed at height *h* (m), *V*_ref_ (m/s) is the known wind speed in reference height *h*_ref_ (m), and the exponent (*α*) is an empirically derived coefficient that varies dependent upon the direction shift or surface roughness which can be determined by (3) [13]

(3) $$\begin{array}{c} \alpha =0.096\left(\log_{10}Z_{0}\right)+0.016{\left(\log_{10}Z_{0}\right)}^{2}+\;0.24, \end{array}$$

where *Z*_0_ is the surface roughness lengths (Table 1).

Wind speed increases with increasing the height above the ground, the pressure and temperature decrease with increasing height, and so the ability of turbine can be affected (see (4) and (5)). Therefore, the height should not be so high to lead to sensible changes in turbine efficiency.

Gross energy production is the total annual energy produced by the wind energy equipment, before any losses, at the wind speed, atmospheric pressure, and temperature conditions at the site. Gross energy production *E*_*G*_ is calculated through [14]

(4) $$\begin{array}{c} E_{G}=E_{U}c_{H}c_{T}, \end{array}$$

where *E*_*U*_ is the unadjusted energy production, and *c*_*H*_ and *c*_*T*_ are the pressure and temperature adjustment coefficients. *c*_*H*_ and *c*_*T*_ are given by

(5) $$\begin{array}{c} c_{H}=\frac{P}{P_{0}},\\ c_{T}=\frac{T}{T_{0}}. \end{array}$$

In these relations, *P* and *T* are pressure and temperature in *H* height, respectively. *P*_0_ and *H*_0_ are standard pressure (101.3 kPa) and standard temperature (288 K), respectively [15].

### 2.3. Wind Speed Distribution in Certain Period

Considering the perpetual changes of wind vector, its analysis based on numeral value as monthly average cannot be exact. For analysis and determination of wind speed, wind direction, and wind speed distribution over a certain period of time, the “Density Function” can be used.

The density function can model the wind speed distribution by using mathematical functions (Pearson, two parameteric Weibull distribution—Log—Logistics, etc.) over a certain period. Among these mathematical functions, the two parameteric Weibull distribution is the most popular function for description of wind speed data. Since in Divandareh station the seasonal average wind speed in spring and summer is greater than in other seasons so we studied their histograms (spring and summer) in different heights. But in Qorveh station, the winter and spring seasons have higher average wind speed compared with others. Therefore, these seasons were considered for studying in 30 and 60 m heights in Qorveh station.

### 2.4. The Weibull Probability Density Function

The Weibull probability density function is defined as

(6) $$\begin{array}{c} f\left(V\right)=\frac{k}{c}{\left(\frac{V}{c}\right)}^{k-1}e^{-{\left(V/c\right)}^{k}}\;\left(k\;>\;0,\;V>\;0,c>1\right), \end{array}$$

where *f*(*V*) is the velocity (*V*) probability, *c* (m/s) is the scale parameter, and *k* (dimensionless) is Weibull shape parameter.

The high *k* value causes the sharpness and the low *k* value causes the broadening Weibull peak. To draw the Weibull probability density function curve, the *k* and *c* values must be determined. One of the determination methods of these two factors is maximum likelihood method. In this method, first by helping of a recursive relationship (see (7)), the *k* value is calculated. Then by using (8), the *c* coefficient is obtained. Consider

(7) $$\begin{array}{c} k={\left[\frac{\sum_{j=1}^{N}V_{j}^{k}LN(V_{j})}{\sum_{j=1}^{N}V_{j}^{k}}-\frac{\sum_{j=1}^{N}LN(V_{j})}{N}\right]}^{-1}, \end{array}$$

(8) $$\begin{array}{c} c={\left(\frac{1}{N}\overset{N}{\underset{j=1}{\sum }}V_{j}^{k}\right)}^{1/k}, \end{array}$$

where *V*_*j*_ is the speed of *j*th sample, *N* is the sum of samples, and (8) is a recursive relationship which needs a primary *k* value that is usually assumed to be equal to 2. But the true primary *k* value can be obtained by (9), [11, 16]. Consider

(9) $$\begin{array}{c} k={\left(\frac{\sigma }{\overset{-}{V}}\right)}^{-1.086},\;1\leq k\leq 10, \end{array}$$

where $\overset{-}{V}$ is mean and *σ* is standard deviation of the samples. The $\overset{-}{V}$ and *σ* values can be calculated from (10), [11]. Consider

(10) $$\begin{array}{c} \overset{-}{V}=\frac{1}{N}\overset{w}{\underset{j=1}{\sum }}M_{j}V_{j},\\ \sigma =\sqrt{\frac{1}{N-1}\overset{N}{\underset{j=1}{\sum }}{\left(V_{i}-\overset{-}{V}\right)}^{2}\;}, \end{array}$$

where *N* is the total number of observations during measurement, *M*_*j*_ is the total number of observations for *V*_*j*_ velocity, and *w* is the number of different velocities.

### 2.5. Weibull Cumulative Distribution Function

The Weibull cumulative distribution function is defined as [11]

(11) $$\begin{array}{c} F\left(V\right)=\;1-e^{-(V/c)k}. \end{array}$$

### 2.6. Rayleigh Probability Density Function

When in the Weibull probability density function *k* = 2, the Rayleigh probability density function can be obtained. It is one of the most applicable functions in this field and can be defined as

(12) $$\begin{array}{c} f\left(V\right)=\;\frac{2V}{c^{2}}e^{{-V}^{2}/c^{2}}. \end{array}$$

The relation between parameter *c* and data average (*μ*) is defined as [17]

(13) $$\begin{array}{c} \mu =\frac{c\sqrt{\pi }}{2}. \end{array}$$

With an expression of the Gamma function Γ(*x*), the average wind speed can be expressed as a function of *c* and *k*, at 10, 30, and 60 m as follows:

(14) $$\begin{array}{c} \Gamma \left(x\right)=\overset{\infty }{\underset{0}{\int }}y^{x-1}\times e^{-y}\;dy, \end{array}$$

where *y* = (*V*/*c*)^*k*^ and *V*/*c* = *y* ^*x*−1^; *x* = 1 + 1/*k*.

The average speed $\overset{-}{V}$ can be calculated by

(15) $$\begin{array}{c} \overset{-}{V}=f\left(k,c\right)=c\Gamma \left(1+\frac{1}{k}\right). \end{array}$$

From (14) and by helping of power law and gamma function, the speed average can be calculated and compared with PDF way value [17].

### 2.7. The Most Probable Wind Speed

The most likely wind blowing and wind speed at special location indicate the usual wind and the usual wind speed distribution in this location. It can be determined by the help of Weibull shape, scale parameters, and following equation [11]

(16) $$\begin{array}{c} \;V_{\text{mp}}\;=c\;{\left(1-\frac{1}{k}\right)}^{1/k}\;\left(\text{m}/\text{s}\right). \end{array}$$

### 2.8. Maximum Wind Energy

It is known that the wind and air stream produce and transport energy.

The maximum of this energy can be determined by (17) and Weibull parameters [11]. Consider

(17) $$\begin{array}{c} V_{\text{ME}}\;=c\;{\left(1+\frac{2}{k}\right)}^{1/k}\;\left(\text{m}/\text{s}\right). \end{array}$$

### 2.9. Wind Power Density (WPD)

One of the important factors to select a place for installation of turbine is wind power density of this place. Without considering the turbine type, one can calculate the annual power of an area by equation [11, 18] as follows:

(18) $$\begin{array}{c} \text{WPD}=\;\frac{P}{A}=\overset{N}{\underset{j=1}{\sum }}V_{j}^{3\;}\rho \frac{1}{N}.\; \end{array}$$

Also the wind power density can be determined by Weibull function parameters and the relation [10]:

(19) $$\begin{array}{c} \frac{P}{A}=\overset{\infty }{\underset{0}{\int }}\frac{1}{2}\rho V^{3}F\left(V\right)\;dV=\frac{1}{2}\rho c^{3}\Gamma \left(\frac{k+3}{k}\right). \end{array}$$

## 3. Results and Discussion

### 3.1. Location, Kurdistan

It is clear that for wind energy production, the place of turbine installation should have high average wind speed and continuous wind blowing. So the speed and other wind parameters studies are considered as initial and important stages of potential evaluation of one region for installation of wind house power [3]. The time interval value to determine the periodical change of wind status is important. The meteorologists believe that 30-year period is needed for long time determination of weather condition and atmospheric changes, and for achieving yearly average wind speed at least 5-year period is needed.

Kurdistan province is located in the west of Iran and the latitude and the longitude of Kurdistan are 35°33′N and 47°08′E, respectively. It has widespread continental mountains and deserts. There is a 2400 m difference between the lowest altitude (A lot in Baneh 900 m) and the highest point (Shahoo with 3300 m). The number of glacial days in a year is 109 and the annual rainfall in normal condition is about 500 mm. This province is situated on path of warm and humid air of the Mediterranean. The dominant winds of this region are western stream from the Atlantic Ocean and the Mediterranean Sea in winter and northwest stream in summer. There are eight weather stations in Kurdistan that record the weather data since 1968 [9]. Also, the new energy organization of Iran (SANA) has established three weather stations in Divandareh (2006), Qorveh (2008), and Zarineh (2011) [1].

**Figure 1:**
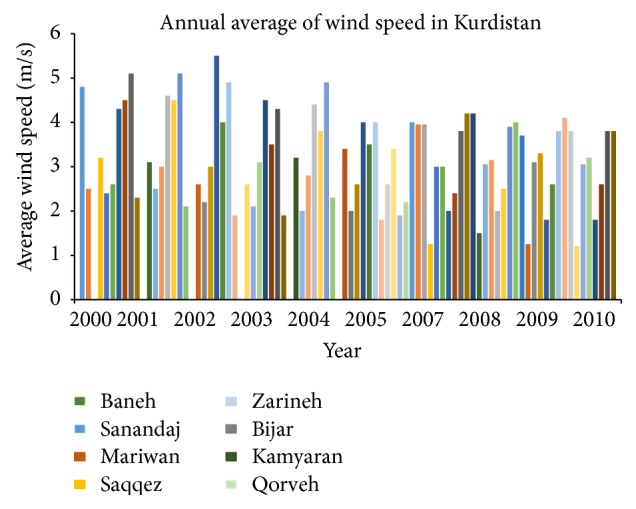
Comparison of yearly average in synoptic stations of Kurdistan at 10 m height.

The curves of monthly average speed and the maximum speed for Qorveh station in period of 2000–2010 were analyzed and the results of this investigation are shown in Table 2, which shows the variation range of monthly average and maximum speed in each station in period of 2000–2010, 10 m in height.  Figure 1 compares the annual average value in Kurdistan Meteorological stations from 2000 to 2010, 10 m in height.

Table 3 shows the monthly average of wind speed in period of 2000–2010 at Kurdistan Meteorological Center, 10 m in height. The wind speed varies in different seasons and months. The wind speed is increased in spring and winter in all stations. The monthly and annual average wind speed in sites of Saqqez, Sanandaj, Marivan, and Kamyaran are lower than the other stations [19, 20].

Table 4 shows the ranking of meteorological stations of the country in period of 10 years (1993–2002) and it is gathered for calibration of wind map to have a reliable picture of wind regime; as can be seen the Zarineh is placed in sixth and Bijar is placed in thirteenth of this ranking at country level [3].

**Figure 2:**
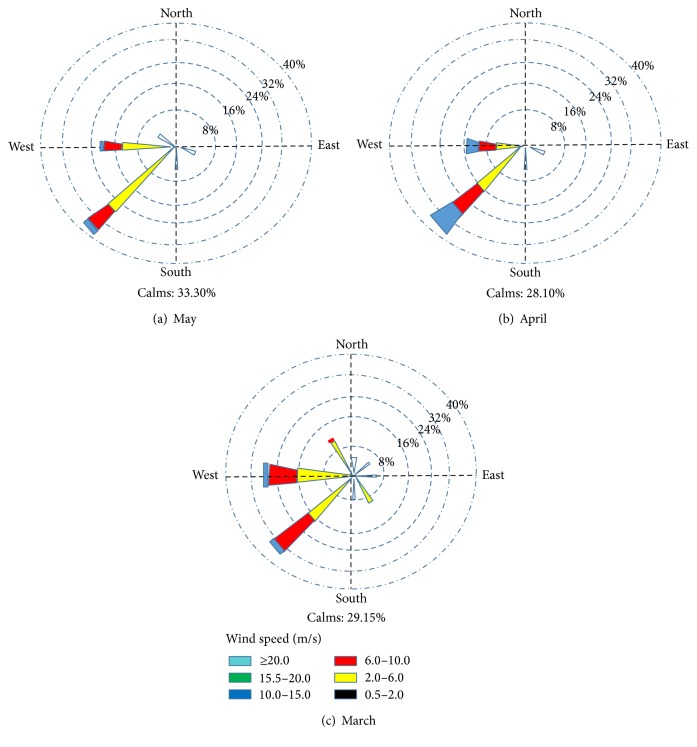
Frequency of wind speed in Qorveh 1998–2007 (Wind Rose).

Figure 2 shows the wind rose at Qorveh station in 1998–2007 in March, April, and May.  Table 5 shows the percentage frequency and wind distribution in different speed ranges in March, April, and May in Qorveh.

**Figure 3:**
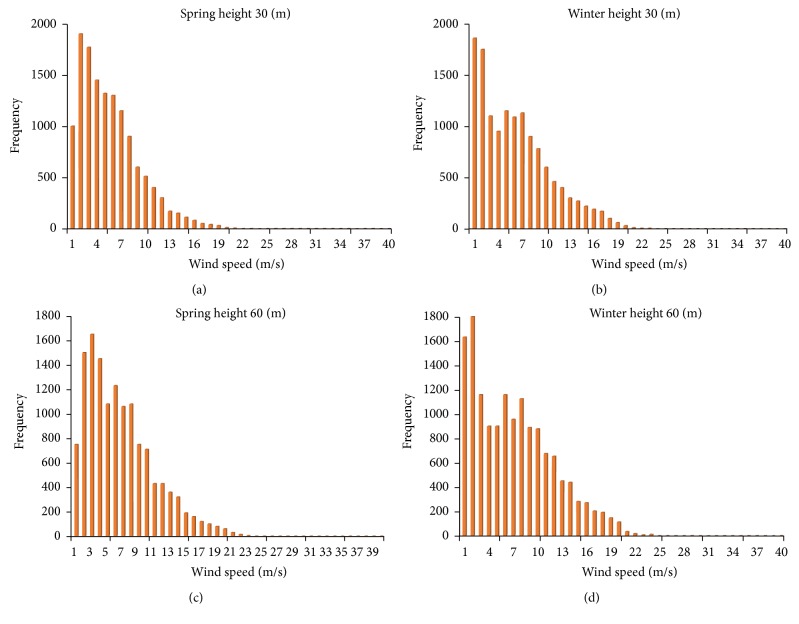
(a) The frequency distribution of wind speed at Qorveh site in spring (height 30 m), (b) frequency distribution of wind speed in Qorveh site in winter (height 30 m), (c) the frequency distribution of wind speed in Qorveh center in spring (height 60 m), and (d) the frequency distribution of wind speed in Qorveh site in winter (height 60 m).

- Based on average and maximum wind speed obtained from Kurdistan meteorological stations in the years 2000–2010, the Baneh, Zarineh, Bijar, and Qorveh centers are in the acceptable range of wind speed potential for wind energy production.
- Based on monthly average values of wind speed in period of 2000–2010, the Baneh, Zarineh, and Bijar sites have higher ranking levels. The maximum wind speed was obtained in spring and winter (Feb, March, April, and May). According to the annual diagram of wind speed of all stations in period of 2000–2010 (not shown), the wind speed variation shows descent trend in all of sites except Qorveh.
- Also in ranking of weather station of Iran, based on average wind speed 10 m in height, during ten years (from 1993 until 2002 which was performed by IRIMO), the Zarineh with average wind speed of 5.8 m/s is at sixth and the Bijar with average wind speed of 5.2 m/s is at thirteenth stage of this ranking. These results confirm that Zarineh and Bijar sites have acceptable wind speed potential.
- According to the monthly Wind Rose diagram, the directions of predominant winds of Zarineh were west and southwest, of the Bijar was south, and of Qorveh were west and southwest (Figure 3).

### 3.2. Wind Speed Variations in Height

The wind speed changes with the change of height above the ground, as the wind speed increases linearly with increasing height above the ground. The wind speed at altitude 450 m is about 4-5 times of its speed on the ground. To obtain more power and efficiency from wind turbines and to access a suitable wind speed, the turbines are installed at high height. The wind speed and direction data at height 10 m from the ground were recorded, where the altitude of turbines installation was higher than that. One of the practical methods for wind parameters measurement at high altitude is using weather masts (installation of several sensors in heights 30, 40, 60, and 80 m). In addition to the mentioned method, there are two important equations to the theoretical calculation of the wind speed in other heights related to a reference wind speed (e.g. 10 m) [18]. Since the wind data of synoptic centers has been recorded at height 10 m. To calculate the wind speed at other heights by theoretical method, some valid regional and local indexes and parameters are needed. Therefore, we use 10-minute data of Qorveh and Divandareh sites in 2007 and 2009, which were recorded by SANA anemometer (the wind speeds were recorded at heights 10, 30, and 40 m and the wind directions were recorded at height 30 and 37.5 m) [1, 3].

### 3.3. Determination of Wind Speed in Three Different Heights

For determination of wind speed by (2) at height (*h*) and reference height (*h*_ref_), the definite *α* coefficient is needed. The coefficient can be determined by the two ways below.

*(i) The First Method for Determination of α Coefficient*.  The surface roughness coefficient (*Z*_0_) can be extracted from Table 1, and *α* can be calculated according to (3).

We determined *Z*_0A_ = 0.25 mm for Divandareh site (A) and *Z*_0B_ = 0.15 mm for Qorveh site (B). These values are determined based on the types of land surfaces. The corresponding *α* coefficients can be determined as [18–23] the following:

(1) If *Z*_0A_ = 0.25 mm → *α*_A=0.188_
(2) If *Z*_0B_ = 0.15 mm → *α*_B=0.15_

*(ii) The Second Method for α Coefficient Determination*. Reverse engineering is used in this method for *α* coefficient determination. The *α* coefficient is calculated by (2) and by the comparison of wind speed (recorded by weather station for each month) in heights 30 and 10 m. The mean value for 12 months is considered as *α* coefficient and the mean values for Divandareh and Qorveh are

(20) $$\begin{array}{c} \alpha_{\text{A}}=0.2,\;\;\alpha_{\text{B}}=0.144. \end{array}$$

Since the *α* coefficient obtained by the second method is determined by sensors and anemometer, the results are close to real values, therefore for wind speeds calculation at other heights the *α* value was considered as *α*_B_ = 0.144 and *α*_A_ = 0.2.

The wind speed at heights 10 and 30 m is determined from excel data and at 60 m determined according to (2) and *α* coefficient. Also, the maximum wind speed in 30 m height was determined. Because of facile installation of domestic 660 and 300 kW turbines at 60 m height, this height was selected for work. The range of wind speed in height 60 m at the Qorveh station was 4.4–7.80 m/s and at the Divandareh station was 4.4–7.7 m/s. The highest values of wind speed were obtained in February, March, and April at these stations.

Also, the maximum wind speed in height 30 m was variable from 17.0 to 26.7 m/s in the Qorveh station and this range for Divandareh station was from 14.2 to 22.7.

Figure 3 shows the frequency distribution diagrams of wind speed obtained in spring and winter in heights 30 and 60 m in Qorveh station.

The frequency for wind speed of 5.5 m/s in spring season in 30 m height is about 1320, but for winter, this number is about 1200 that is lower than that of spring season.

The velocity probability *F*(*V*), the scale parameter *c* (m/s ), and the Weibull shape parameter *k* (dimensionless) which are obtained with MATLAB, are shown in Table 6.

The power density of Qorveh, a site in two heights of 30 and 60 m, has been calculated, results of which are shown in Table 7. The basis of this calculation was ten-minute data.

### 3.4. The Type of Turbine

To select a suitable turbine for a certain place, many factors must be considered. One of the most important factors is the wind speed at this place. As regards, the monthly average wind speed in Qorveh station in 60 m height and technical study of the various turbines from different companies (such as GE, Suzlon, Norder, Vestas, and Gamesa), because of high capacity, facile installation, and ability to produce power according to site condition (compatibility with region) the turbines from Gamesa (G58-850) and Suzlon (S64-650) companies were selected.

## 4. Conclusions

In summary, in this work, by statistical analyses of wind direction and speed in years of 2000–2010 in 10 m height, the Qorveh, Baneh, Bijar, and Zarineh stations were recognized as the areas of good wind energy potential. In these areas, the winds blow at high speed and continually in winter and spring. The difference between maximum and minimum monthly average wind speeds was not great.

Investigation shows that there has been a slight declining trendat wind speed in all of these sites during the recent eleven years. In part two of this work, a model for calculation of wind speed in different heights based on wind speed at reference height and surface roughness length is presented.

Based on mathematical model of Weibull density function, the monthly, seasonal, and annual wind speed distribution probabilities were determined.

The results of simulation model with MATLAB are shown as curves, tables, and parameters. The Weibull probability density function curve and the histograms of Qorveh site based on 10-minute period data were drawn at different heights.

Wind power densities of Qorveh and Divandareh sites in two heights of 30 and 60 meters were calculated and their wind class was indicated as 2–5, but their annual wind class was 3. Based on the wind power density data, the Qorveh site was distinguished as a suitable place for turbine installation. Also by the studying of SANA wind Atlas, the Divandare (Zarineh), Bijar, and the regions between Qorveh and Dehgolan, with average wind velocity of 7 to 9 m/s, were determined as places with high wind potential. The results of the technical studies of various turbines from different companies show the G58-850 and SU turbine models are suitable for installation in our sites.

## Figures and Tables

**Table 1:** Surface roughness length for different types of land surfaces.

Rank	Types of land surfaces	Roughness length *Z*_0_ (mm)
1	Very smooth—iced or marshy	0.01
2	Clam and free sea	0.2
3	Turbulent sea	0.5
4	Snowy level and land	3
5	Grassland	8
6	Pasture and tore	10
7	Farm land	30
8	—	50
9	Sparse trees	100
10	Gross trees—building fence with low density	250
11	Jungle	500
12	Vicinity and environs of city	1500
13	City center with high buildings	3000
14	Environment of big cities with high tower	4000

**Table 2:** The average and maximum changes of wind speed at height 10 m for synoptic stations of Kurdistan 2000–2010.

The name of weather station	Average wind speed (m/s) range	Maximum wind speed (m/s) range
Baneh	2.5–6.9	15–26
Bijar	1.9–6.6	15–22
Zarineh	2.5–7.6	15–25
Saqqez	1.2–4.7	17.8–10
Sanandaj	0.7–3.5	10–15
Qorveh	1.33–5.4	17.5–10
Marivan	0.6–3.380	10–17

**Table 3:** Monthly average wind speed of synoptic station of Kurdistan.

Synoptic center	The average of wind speed (m/s) in months for years 2000–2010											
	Jan	Feb	Mar	Apr	May	Jun	Jul	Aug	Sep	Oct	Nov	Dec
Baneh	64.3	98.3	18.5	22.5	5	15.5	65.4	56.4	63.4	11.4	79.3	72.3
Bijar	51.3	44.4	4.5	41.5	46.4	93.2	49.2	42.2	61.2	87.2	26.2	14.3
Zarineh obato	55.3	28.4	58.5	68.5	72.4	28.4	07.4	4	03.4	14.4	49.3	25.2
Saqqez	06.2	55.2	58.3	61.3	92.2	76.2	91.2	8.2	48.2	54.2	87.1	6.2
Sanandaj	92.1	24.2	72.2	62.2	28.2	20.2	29.2	14.2	77.1	66.1	41.1	65.1
Qorveh	62.2	46.2	4.4	24.4	52.2	01.2	9.2	83.2	75.2	9.2	58.2	63.2
Marivan	44.1	45.2	54.2	46.2	89.1	89.1	88.1	82.1	76.1	75.1	22.1	4.1

**Table 4:** Ranking of Iran weather station (measured at 10 m height).

Ranking	Station	E	N	Elevation (m.a.s.l.)	Wind speed (m/s)
1	Manjil	49° 24′	36° 44′	333.0	8.8
2	Zabol	61° 29′	31° 02′	489.2	6.9
3	Eghlid e-fars	52° 38′	30° 54′	2300.0	6.5
4	Bushehr coastal	50° 49′	28° 54′	8.4	6.2
5	Zahak	61° 41′	30° 54′	495.0	6.1
6	**Zarineh**	46° 55′	36° 04′	2142.6	5.8
7	Khoor-birjand	58° 26′	32° 56′	1117.4	5.8
8	Norabad-e-lorestan	48° 00′	34° 03′	1859.0	5.7
9	Ardebil	48° 17′	38° 15′	1332.0	5.7
10	Bandae e dacir	51° 56′	27° 50′	4.0	5.6
11	Sahand	46° 07′	36° 57′	1641.0	5.3
12	Kahnouj	57° 42′	27° 58′	469.7	5.3
13	**Bijar**	47° 37′	35° 53′	1883.4	5.2
14	Khodabandeh	48° 35′	36° 07′	1887.0	5.2
15	Firoozkooh poll	52° 24′	35° 43′	2985.5	5.0
16	Rafsanjan	55° 54′	30° 25′	1580.9	5.0

**Table 5:** Percentage frequency of wind speed in different ranges at March, April, and May, in Qorveh.

Wind speed range (m/s)	Percentage frequency of wind speed in month (%)		
	Mar	Apr	May
Calm wind	51.4	48.7	53.5
2–6 or lower than 2	1.8	19.5	12.5
6–10	7.5	8.0	7.5
10–15	2.0	4.2	2.5
15–20	21.0	19.5	24.0

**Table 6:** The *c* and *k* value in Qorveh site at heights 60, 30, and 10 m determined by PDF way and power law.

Studied period (seasonal annual) (2006–2009)	*k* and *c* parameters in density function of Weibull in three heights											
	60 m				30 m				10 m			
	*c*	*k*	*V*	*V* = *f*(*c*, *k*)	*c*	*k*	*V*	*V* = *f*(*c*, *k*)	*c*	*k*	*V*	*V* = *f*(*c*, *k*)
Spring	6.47	1.5	5.85	5.58	5.52	1.5	5	4.95	5	1.58	4.5	4.42
Summer	5.68	1.51	5.15		4.84	1.5	4.4	4.37	4.51	1.64	4.4	4
Fall	5.74	1.28	5.24	5.24	4.3	1.12	4.12	4.12	3.4	1.2	3.78	3.77
Winter	6.77	1.24	6.35	6.31	5.77	1.24	5.42	5.40	5.28	1.34	4.85	4.72
Annual	6	1.2	5.53	4.42	5.15	1.23	4.74	4.71	4.75	1.44	4.31	4

**Table 7:** Power density for Qorveh site at 30 and 60 m height.

Period of time	Height m	Average wind power density (W/m^2^) at 30 and 60 m height												
		Jan	Feb	Mar	Apr	May	Jun	Jul	Aug	Sep	Oct	Nov	Dec	Annual
2008-2009	30	177.7	352	458	324	206.6	112.6	94	141.4	186	86	173	446	220
	60	287.5	569	739	523	333	182	151.6	228	302	140	280	725	356
